# Electrical resistivity of the Fe–Si–S ternary system: implications for timing of thermal convection shutdown in the lunar core

**DOI:** 10.1038/s41598-022-21904-y

**Published:** 2022-11-08

**Authors:** Joshua A. H. Littleton, Wenjun Yong, Richard A. Secco

**Affiliations:** 1 Department of Earth Sciences, University of Western Ontario, London, ON N6A3K7 Canada; 2 Department of Earth and Planetary Sciences, Washington University in St. Louis, St. Louis, MO 63130 USA

**Keywords:** Solid Earth sciences, Planetary science, Core processes

## Abstract

The composition of the lunar core has been suggested to be Fe-rich with varying amounts of lighter elements, such as Si and S. Presence of Si and S affects electrical and thermal transport properties and thus influences core thermal processes and evolution. Paleomagnetic observations constrain a high intensity magnetic field that ceases shortly after formation of the moon (~ 3.5–4.2 Ga year ago), and thermal convection in the core may contribute to generation of this field. In this study, the electrical resistivity of Fe-14 wt% Si-3 wt% S was measured in both solid and molten states at pressures up to 5 GPa and thermal conductivity was calculated via the Wiedemann–Franz Law from the electrical measurements. The results were used to estimate the adiabatic conductive heat flux of a molten Fe-14 wt% Si-3 wt% S lunar core and compared to a Fe-2-17 wt% Si lunar core, which showed that thermal convection of either core composition shuts down within the duration of the high intensity magnetic field: (1) 3.17–3.72 Ga year ago for a Fe-14 wt% Si-3 wt% S core; and (ii) 3.38–3.86 Ga years ago for a Fe-2-17 wt% Si core. Results favouring compatibility of these core compositions with paleomagnetic observations are strongly dependent on the temperature of the core-mantle boundary and time-dependent mantle-side heat flux.

## Introduction

Determining the transport properties of iron (Fe) and Fe alloys at high pressure (*P*) and temperature (*T*) conditions is crucial for understanding terrestrial core energetics and thermal states^1^. Electrical resistivity (ρ) measurements provide an indirect method to delineate crystal structure, and electronic and magnetic transitions of materials (e.g.,^2,3^). Moreover, in the case of pure metals and alloys, electronic thermal conductivity (κ_e_) can be estimated from the measurements of ρ via the Wiedemann–Franz Law (WFL)^4^. Experimental setups in large volume presses have undergone considerable refinement in recent years that have allowed for reliable measurements of ρ and by extension calculated estimates of κ_e_ in both solid and liquid states^5–7^. Results of these experiments are used to evaluate terrestrial core conductive heat flow, as well as permissible mechanisms of convection that may take place in liquid or partially molten portions of these cores. These processes also have implications for terrestrial bodies that currently produce, or once believed to have produced, an internally generated magnetic field since core convection is necessary to drive and sustain a core dynamo^1^.

There have been a considerable number of experimental and theoretical studies conducted over several decades investigating ρ and κ_e_ of Fe at high *P* and *T* (e.g.,^2,8–12^) because it is the dominant element comprising terrestrial cores. Lighter alloying or core constituent elements, such as sulphur (S), silicon (Si), oxygen, carbon, and hydrogen, are also suggested to be present and required to account for core density deficits and geochemical mass balancing^13,14^. Similar investigations extending to binary Fe-LE systems, where LE is a candidate core light element (e.g. S, Si), have been conducted for some time with a noticeable increase of investigative focus in the last two decades in an effort to better constrain values of ρ and κ of terrestrial cores^3,11,12,15–24^. Since more than one LE is likely to be present in a terrestrial core (e.g.,^25^), studies of ternary Fe–LE_1_–LE_2_ systems are a necessity; albeit, comparably fewer investigations have been conducted at high *P* and *T* (e.g.,^26–30^). While it is well known that the presence of LE is expected to hinder the propagation of heat-carrying conducting electrons in the metal alloy, thus increasing ρ and decreasing κ_e_ compared to pure Fe, the magnitude of this impurity effect on these transport properties remains ill-constrained, adding to the imperative for more experimental measurements.

Paleomagnetic studies have indicated the Moon had a core dynamo-produced magnetic field in the past and that the lunar dynamo may have persisted up to 1.92–0.80 Ga ago, suggesting that convective motions in a liquid Fe-alloy outer core terminated relatively recently^31^. However, the duration of lunar dynamo action, and the convection mechanisms that drive the generation of the field, are strongly debated. Observations of a high intensity magnetic field and its duration are better constrained, with a duration ranging between 4.2 and 3.5 Ga ago^32–34^. This high intensity field generated by the lunar dynamo is attributed to vigorous movements in the liquid core^35^ which may have been caused by thermal convection. A prerequisite for whole or upper liquid core thermal convection to occur is that the heat flow through the core-mantle boundary (CMB) must exceed the heat conducted down the adiabat to the core-side of the CMB^1^. Once this condition is no longer satisfied, thermal convection is shut down and convective motions of the liquid core are reliant on other mechanisms such as mantle or whole body precession, or solidification and growth of the inner core^32–36^. Since the effect of the presence of LE in the core is to lower κ_e_, which is a parameter directly proportional to adiabatic conductive heat flow, the duration of the high intensity field and shutdown of thermal convection may be considered an additional constraint on lunar core composition. If κ_e_ is too strongly dampened by LE content, and provided that the heat flow through the CMB as a function of lunar age is known and assumed to decrease with time, the estimated age of thermal convection shutdown and end of the high intensity field will be younger (i.e. last longer), outside the duration bound, and thus incompatible with observations.

Si and S as candidate light elements in the lunar core have been argued on the basis of sound velocity and geodetic data, and meteorite geochemistry [e.g.]^37,38^. S has a greater siderophilic character and its presence in an Fe-rich core depresses the liquidus freezing point more than Si^39,40^. Given the ill-constrained core *T* through time and at the present day, S permits a liquid outer core to exist within a wider range of *T*. In this study, we experimentally measured the ρ of Fe-14 wt% Si-3 wt% S up to 5 GPa and *T* up to ~ 2000 K. Henceforth, compositions will be reported in a notation style where the numerical values indicate wt.% of a LE (e.g., Fe_83_Si_14_S_3_). The Fe alloy composition and experimental *P*–*T* conditions are directly relevant to the core of the Moon (~ 4.5–5.5 GPa, ~ 1470–1870 K)^14,35,37,41–45^. The measurements were used to calculate κ_e_ using the WFL and the lunar adiabatic heat flux of an Fe–Si–S core was determined from the calculated estimates of κ_e_. Core-side adiabatic heat flux was compared to models of age-dependent heat flow through the CMB and thermal convection shutdown ages were estimated.

## Results

### Post-experiment sample analyses

**Figure 1:**
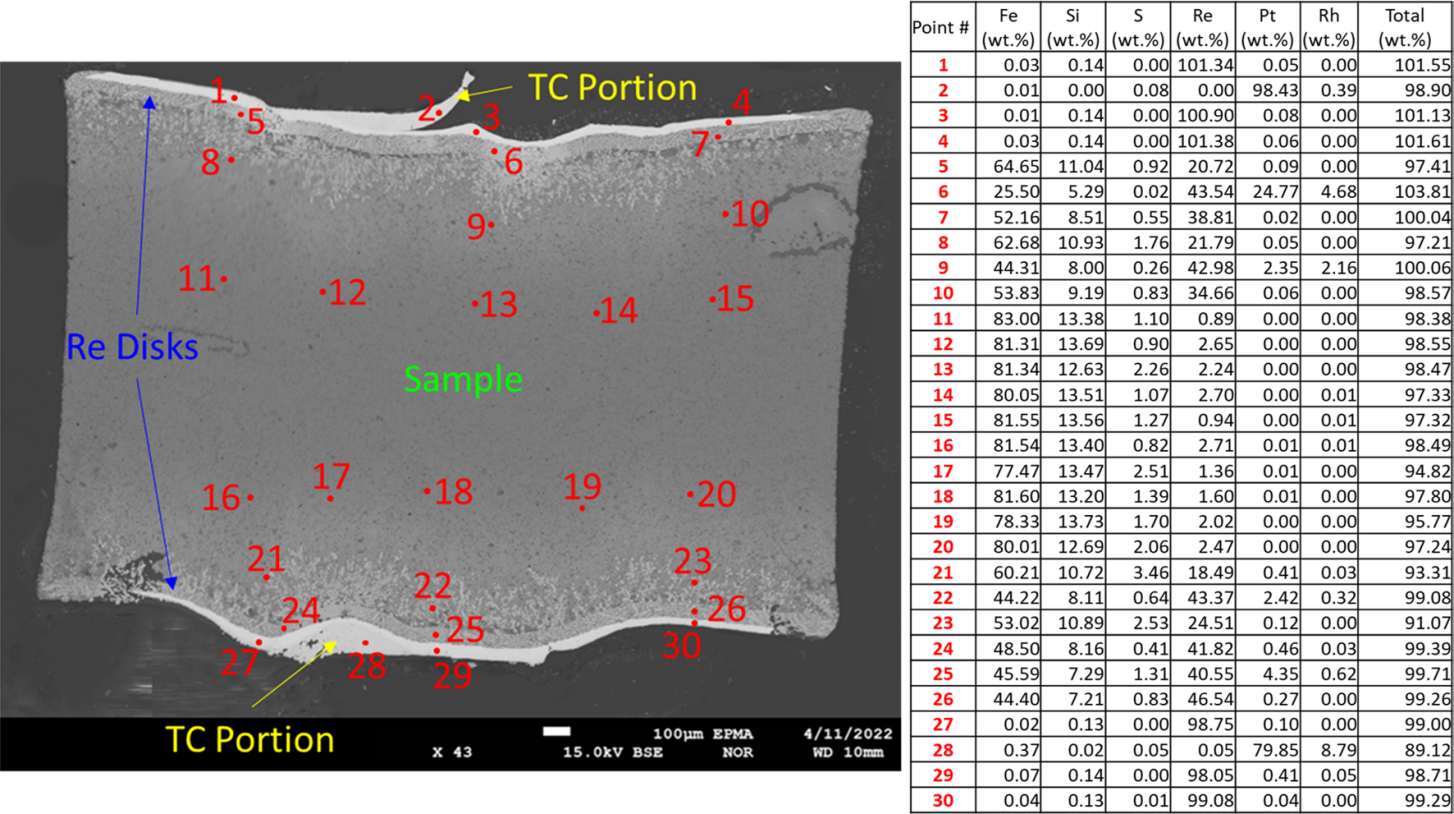
Backscattered electron image of a cross-section of the 4 GPa sample extracted from the pressure cell post-experiment and -heating up to ~ 1980 K, ~ 300–400 K above expected melting temperatures. Portions of the thermocouple (TC), rhenium (Re) disks, and bulk sample have been annotated alongside microprobe locations. Results of the microprobe have been tabulated.

Figure 1 displays a backscattered electron image of the cross-section of the 4 GPa sample recovered from the three-sectioned cubic *P* cell (see “Methods”) after an experiment. Tabulated electron microprobe results of 30 locations correspond to the labeled sites in the adjacent image. The analyses showed appreciable rhenium (Re) contamination within the sample near the contacts of the Re disks. Re content in the sample decreased away from the contacts towards the center, approaching more modest values of up to a couple weight percent. Contamination of molten Fe-rich samples using similar *P* cell designs with refractory metal disks has been consistently observed and thus was expected in our experiments [e.g.]^2,3,5,6,18,20,22,24,46^. A contributing factor to sample contamination and apparent whole-sample distribution of Re observed from the microprobe analyses is the significant elevation of the measurement *T* above the expected liquidus *T*^39^ by ~ 300–400 K. To compare sample contamination of Re as a function of *T*, an experiment was conducted at 4 GPa up to ~ 1650 K. Figure S1 in Supplementary Information displays a backscattered electron image of this *P* cell centered on the sample after the experiment alongside tabulated electron microprobe results of 26 locations correspond to the labeled sites. Expectedly, most sample contamination by Re occurs nearest to the disk contacts. In contrast, because of the significantly reduced measurement *T*, Re content within bulk of the sample was either below the detection limit or not detected. We note, however, that the presence of Re in the bulk of the sample acts as an additional impurity. Matthiessen’s rule suggests that the impurity contribution to total resistivity remains constant and therefore on an increasing ρ with T in the liquid, the impurity effect becomes reduced. Moreover, the study by Littleton et al.^47^ showed that at the liquid T of the current study, the resistivity of a rod of pure Re is 85–93 μΩ cm, or only ~ 23% of the total resistivity of our sample. Nevertheless, while the effect of Re on total resistivity is expected to be small at very high *T* in the liquid state^47^, the values of ρ of a molten sample reported in this work should be treated as upper boundary values.

### Electrical resistivity and thermal conductivity

**Figure 2:**
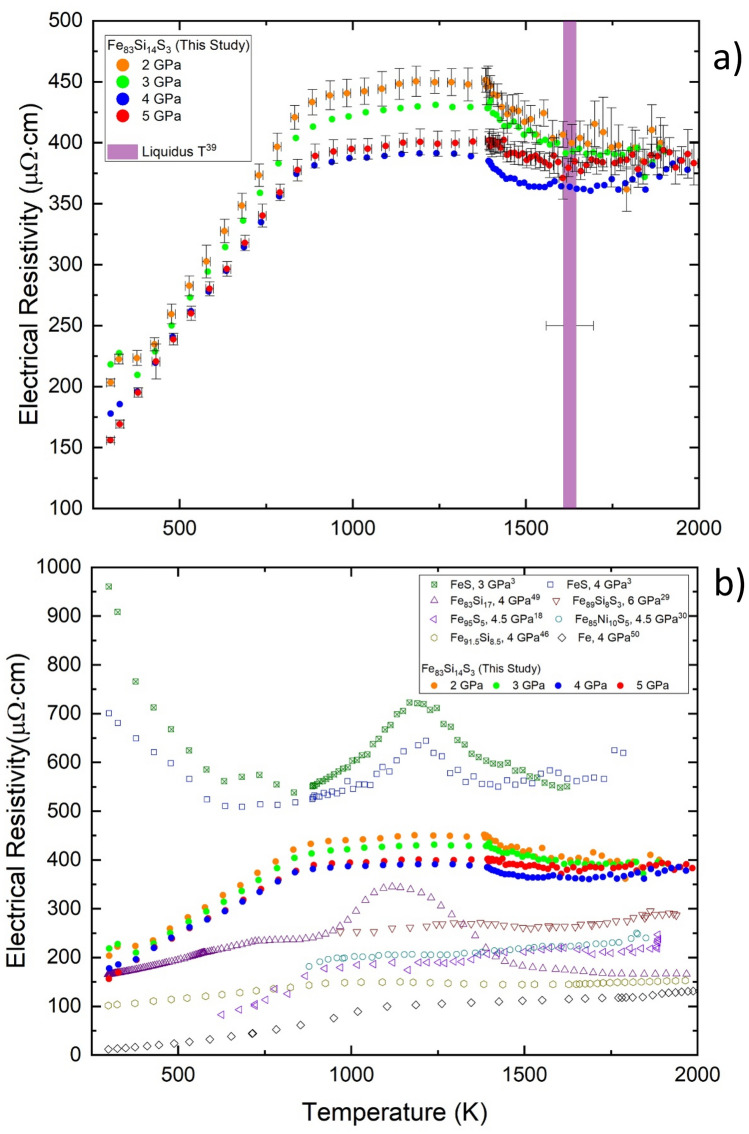
(**a**) Measured electrical resistivity of Fe_83_Si_14_S_3_ at pressures of 2–5 GPa (± 0.2 GPa) as a function of temperature. Error bars of *T* and ρ are shown for all 2 and 5 GPa measurements and are representative for all measurements displayed in the figure. Extrapolated pressure-dependent liquidus temperature estimates of a similar composition within the Fe–Si–S system (Fe_88_Si_4_S_8_)^39^, are shown by the vertical line spanning the height of the vertical axis. The width of the line corresponds to estimated liquidus temperature from 2 GPa (left side) to 5 GPa (right side) with an uncertainty of ± 50 K. (**b**) Measured electrical resistivity values of Fe_83_Si_14_S_3_ from this study are compared to several previous studies of similar or relevant compositions^3,18,29,30,46,49,50^.

Figure 2a shows measured values of ρ of Fe_83_Si_14_S_3_ up to 5 GPa and ~ 2000 K from this study, and Fig. 2b compares the results of this study to other works with similar or relevant Fe-alloy compositions. In general, the effect of increasing *P* decreased ρ while the effect of increasing *T* increased ρ (Fig. 2a). These trends are typical for metals and Fe-alloys with relatively low impurity contents (e.g.,^2,46^). As shown in Fig. 2b, Fe_83_Si_14_S_3_ was observed to have significantly larger ρ compared to pure Fe and is attributed to well-known impurity effects. Similarly, the ρ of Fe_83_Si_14_S_3_ was larger than that of Fe_95_S_5_^18^, Fe_91.5_Si_8.5_^46^, Fe_89_Si_8_S_3_^29^, and Fe_85_Ni_10_S_5_^30^ at all *P* and *T*, and all of these alloys also showed larger ρ than pure Fe. Investigations on Fe–Ni alloys with compositions typically expected for terrestrial cores (0–10 wt% Ni; e.g.,^19,24,48^) have shown that the effect of Ni on ρ is small. Thus, in comparison to Fe_85_Ni_10_S_5_, the S content is mainly responsible for differences in ρ. With respect to *T*-dependence of ρ, as well as LE content and *P* effects, our results are in excellent agreement with Fe_89_Si_8_S_3_. Our results of ρ were expected to be larger than Fe_89_Si_8_S_3_ because of our greater LE content and lower P conditions by comparison with the net effect being a less electrically conductive metallic sample and thus increased ρ. In contrast, the ρ of Fe_83_Si_14_S_3_ is significantly lower than our recent results on FeS. We note, however, there is considerable disagreement in reported experimentally measured values of ρ for FeS at high *P* and *T* by different groups^[3]^(references therein). The values of ρ for FeS shown in Fig. 2b are the lower-bound values and allow for appropriate figure scaling.

**Figure 3:**
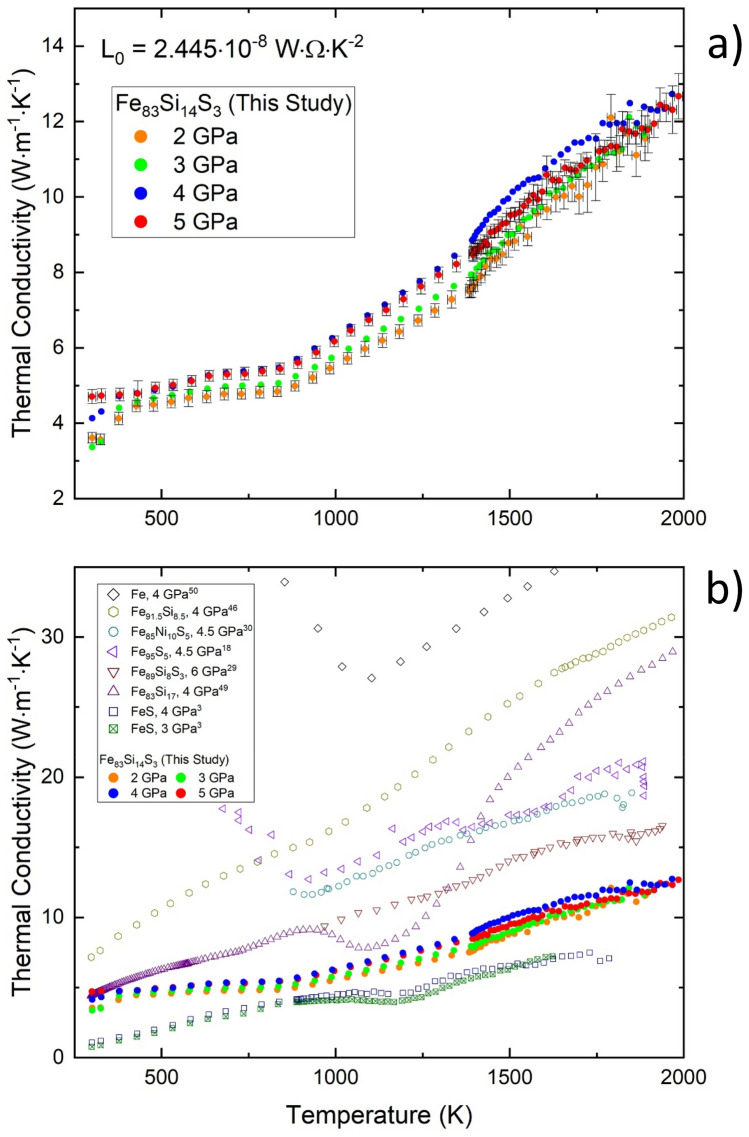
(**a**) Calculated electronic thermal conductivity of Fe_83_Si_14_S_3_ at pressures of 2–5 GPa (± 0.2 GPa) as a function of temperature. Error bars of *T* and κ_e_ are shown for all 2 and 5 GPa measurements and are representative for all measurements displayed in the figure. (**b**) Calculated electronic thermal conductivity of Fe_83_Si_14_S_3_ from this study compared to several previous studies of similar or relevant compositions^3,18,29,30,46,49,50^. Low temperature values of electronic thermal conductivity of Fe (up to ~ 62 W m^−1^ K^−1^) are not shown for figure scaling purposes.

Total thermal conductivity (κ) is the sum of phononic and electronic (κ_e_) contributions. The conduction electrons of electrical conductors such as pure Fe and Fe alloys are the dominant carriers of both charge and heat. In these metallic conductors, κ_e_ is the dominant contribution to the total κ such that κ ≈ κ_e_. The WFL, shown in Eq. (1), relates *T* and ρ to κ:

1 $$\upkappa \, \approx \,\upkappa _{{\text{e}}} = {\text{ L}}_{0} T/ \,\uprho$$

where L_0_ is the Sommerfeld value (2.445 10^–8^ WΩ K^−2^) of the Lorenz number and is considered a constant term^4^. This method of using measurements of *T* and ρ to determine κ_e_ (or κ) is more convenient and less challenging than direct measurements of κ that are highly reliant on well-controlled *T* gradients. Figure 3a shows calculated values of κ_e_ of Fe_83_Si_14_S_3_ up to 5 GPa and ~ 2000 K from this study alongside liquidus *T* estimates, and Fig. 3b compares the results of this study to the other studies shown in Fig. 2b. In general, the effect of increasing *P* and *T* increased κ_e_ (Fig. 3a). The effect due to LE content is easily observed and the trends with respect to composition are a contrasting counterpart to ρ: whereas increasing LE content increased ρ Fig. 2b), increasing LE content decreased κ_e_ (Fig. 3b).

## Discussion

The larger near-room *T* values of ρ at 2 and 3 GPa compared to 4 and 5 GPa (Fig. 2a) is due to the stability of FeS I (troilite)^51^, a semi-conducting material. The FeS content was not large enough to significantly alter *T*-dependent behaviour (i.e. decreasing ρ with increasing *T*) and is masked or dominated by metallic behavior. After a small increase in *T*, FeS I underwent a phase transition to FeS IV (hexagonal)^51^, which is a more electrically conductive semi-conducting material, and ρ decreased. At 4 and 5 GPa, FeS II (MnP-type) or FeS IV is the stable phase at room *T*. The former phase has not been consistently observed in studies investigating FeS phase relationships^51,52^. Our results are incapable of distinguishing between the two phases as noted previously with a similar experimental setup^3^. The phase transition from FeS IV to FeS V (NiAs-type)^51^ at ~ 650 K, the latter having metallic behaviour, cannot be discerned. The distinct change and shallowing in the *T*-dependent trend of ρ around ~ 850 K for all *P* is attributed to the ferromagnetic–paramagnetic transition of Fe (~ 1043 K for pure Fe at ambient *P*). The Curie *T* decreases with increasing Si content in Fe at ambient *P*^53^ and high *P*–*T* investigations of Fe have shown the Curie *T* decreased with increasing *P* (e.g.,^2^). Thus, the lower *T* of our observed magnetic transition is attributed to both LE content and *P* effects. Moreover, the magnetic transition observed in our results is also in good agreement with Fe_95_S_5_^18,30^ and Fe_85_Ni_10_S_5_^30^, suggesting S may have a similar effect as Si. As well, with increasing Si content, the *T*^2^-dependence in the ferromagnetic region lessens compared to pure Fe. For instance, Fe_91.5_Si_8.5_ displayed a weak *T*^2^-dependence^46^ while a linear *T*-dependence was observed in Fe_83_Si_17_^49^. Fewer measurements over a small *T* range in the ferromagnetic region and measurement scatter obscure any well-defined trend in Fe_95_S_5_^18^—a linear *T*-dependence fit is as good as a *T*^2^-dependence fit. However, a *T*^2^-dependence is greater than for pure Fe and suggests greater spin-disorder scattering enhanced by presence of FeS V. Our results show a linear *T*-dependence in this *T* region and indicate that low S contents provides the same *T*^2^ weakening effect as increasing Si content.

Within the *P* range of 20–80 GPa and sub-solidus *T*, the crystal structures of the Fe–Si–S system with LE contents expected for terrestrial cores are an Fe-Si alloy (*hcp*) co-existing with an Fe–S compound (Fe_3_S)^39,54^. Presuming a co-existence of separate Fe–Si and Fe–S phases also applied at lower *P*, we consider the *P* and *T* effects on ρ of Fe_83_Si_17_ and FeS, the starting components of our powder sample mixture, separately. Within the experimental *P*–*T* conditions of this work^53^, Fe_83_Si_17_ may undergo *bcc*-sublattice structure changes (α_1_ or DO_3_-type, α_2_ or B2) on heating that differ in long- and short-range Si atomic ordering and *hcp* structure changes (β), although studies on the exact types of structures and stability regimes for the Fe–Si system lack consensus^40^. Nevertheless, at low to moderately high *T*, we are unable to infer from our ρ results any solid state phase transitions. This suggests either that the ρ of the *bcc*-sublattice structures (i.e. α_1_ to α_2_) are indistinguishable, no phase transitions had occurred, or the effects due to the presence of S and FeS phases mute what would be otherwise noticeable changes in ρ behaviour. In the higher *T* region, where measurement and heating rate was increased, the *T*-dependence of ρ becomes negative for an approximate 200 K interval of *T* beginning around 1400 K. In this *T* range, Fe_83_Si_17_ may undergo a phase transition from α_1_ to β^40,53^ while FeS is expected to melt^51^—establishing the solidus of Fe_83_Si_14_S_3_. The decrease of ρ at the start of this *T* interval could be related to the transition to β, implying that this phase is more electrically conductive than α_1_ at high *T*. Using the results of the FeS (~ 35 wt% S^3^) for insight (Fig. 2b), the ρ of liquid is lower compared to values at *T* preceding the solidus and may indicate in our current results that the presence of a molten FeS component (Fig. S2) in the latter half of this *T* interval reduced the ρ of the sample. The decrease of ρ was not observed to be as drastic due to the 1:8 (FeS:Fe_83_Si_17_) proportion of the mixed powder sample. At *T* beyond this interval (≥ 1600 K), the *T*-dependence increased gradually to near *T*-independence with measurement values at all *P* converging to ~ 390 μΩ cm. The increasing *T*-dependence may be attributed to melting of Fe_83_Si_17_ and additional structural disorder. Phase diagrams at ambient and high *P* indicate Fe_83_Si_17_ will undergo partial melting (α_1_-Fe-Si solid + Fe-Si liquid) at *T* just prior to melting^40,53^. However, this phase regime is stable for a small *T* interval (~ 50 K at 1 atm). Since our heating rate was fast all throughout ~ 1400–2000 K (~ 47 s), the *T* interval between the solidus and liquidus of the system was briefly attained but quickly eclipsed within several seconds (~ 5 s). Thus, we considered the bulk contribution affecting the *T*-dependence at this high *T* partial melt region was due to completely molten Fe_83_Si_17_. Liquidus *T* for the Fe_88_Si_4_S_8_ system have been investigated^39^. Extrapolation of the results of that study to 2–5 GPa are shown in Fig. 2a to act as a guide to delineate between liquid and solid-molten states, which cannot be reliably delineated from our measurements of ρ. While the LE contents pertaining to the liquidus curves are opposite to this study, the ± 50 K of these estimates may be enough to span the actual liquidus *T* of Fe_83_Si_14_S_3_ at each *P*.

**Figure 4:**
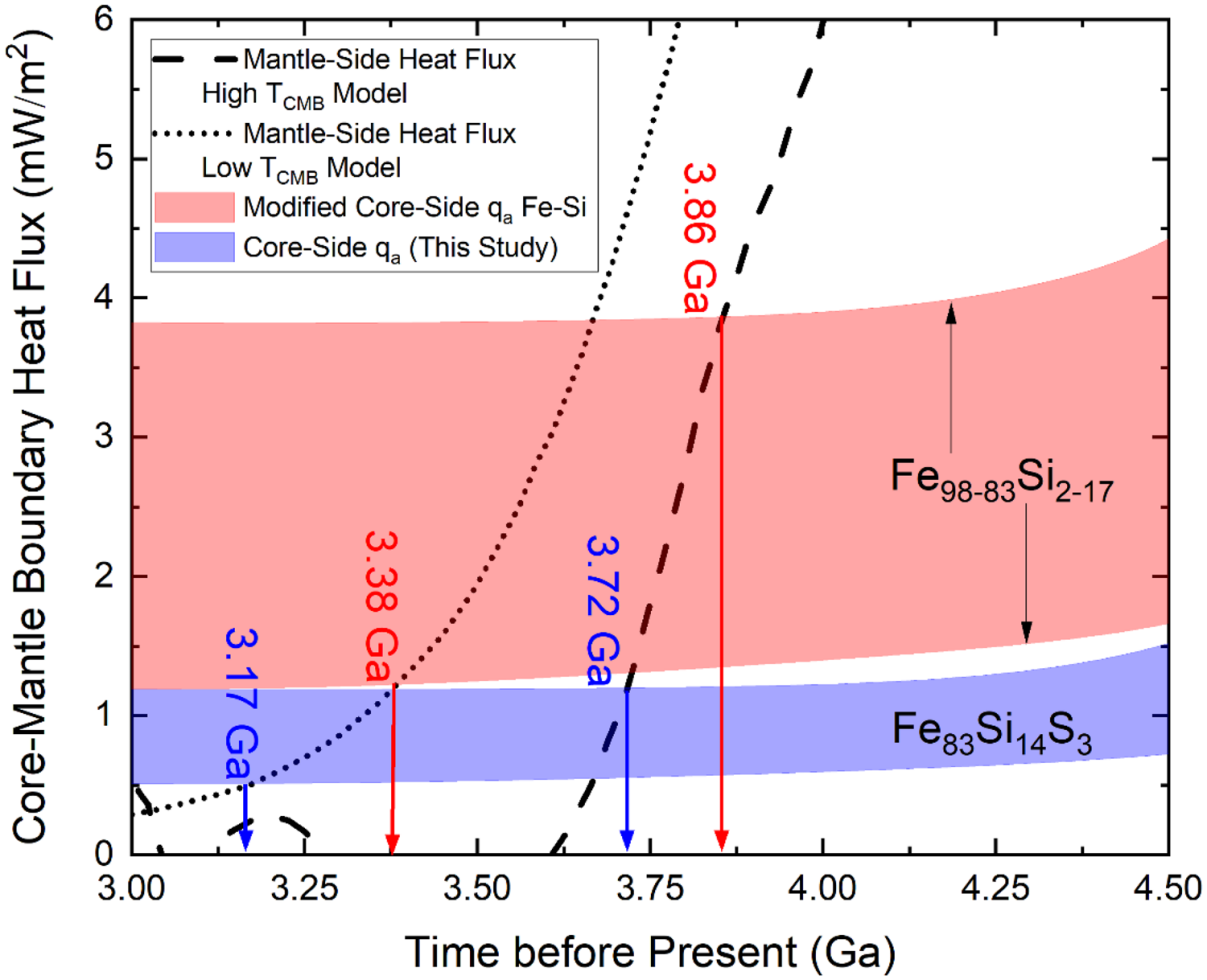
Heat flux at the lunar CMB. Coloured bands annotated with Fe-Si (modified from Berrada et al.^46^) and Fe_83_Si_14_S_3_ (this study) compositions show calculated adiabatic heat flux. The dotted and dashed curves are models of the mantle-side heat flux from Laneuville et al^44^. The vertical arrows show the corresponding range of ages for thermal convection shutdown.

We adopted a similar formulation and calculational procedure as Berrada et al.^46^ and used references therein for parameter values to apply our results to the Moon and determination of the adiabatic heat flux on the core-side of the CMB (q_a_) for our Fe_83_Si_14_S_3_ composition. The adiabatic heat flux was calculated using Eq. (2) below:

2 $${\text{q}}_{{\text{a}}} = - {\upkappa }_{{\text{e}}} \left( {\frac{\partial T}{{\partial r}}} \right)_{a} = {\upkappa }_{{\text{e}}} \left( {\frac{{4\pi rG\alpha_{c} d_{c} T_{CMB} }}{{3C_{P} }}} \right)$$

where *r* is lunar CMB radius (330 km), *G* is the universal gravitational constant, *α*_*c*_ is lunar core thermal expansion (5.25–10.3∙10^–5^ K^−1^), *d*_c_ is lunar outer core density (5.16 g/cm^3^), *T*_*CMB*_ is the lunar CMB temperature, and *C*_*P*_ is the specific heat at constant *P* (800–850 J kg^−1^ K^−1^). Departing from Berrada et al^46^, we allowed *T*_*CMB*_ to vary as a function of lunar age based on the models by Laneuville et al^44^. We focused on two of their models—model 2 and model 5 (their Fig. 8a, b), which we will designate as the low *T*_*CMB*_ model and high *T*_*CMB*_ model, respectively. These two models can be considered boundary models as their other models indicate either similar or intermediate values of *T*_*CMB*_ with increasing age. Similarly, these two models constrain the values of mantle-side heat flux at the CMB with increasing age within our selected time frame to which our results will be compared. As another departure from Berrada et al^46^, we allowed the value of κ_e_ to vary as a function of *T*_*CMB*_ and thus as a function of lunar age. *T*_*CMB*_-dependent values of κ_e_ were either interpolated or extrapolated from our calculated 5 GPa (± 0.2 GPa) results. These adjustments were made to reflect that *T*_*CMB*_ in the past (or interior *T*, in general) was hotter than present-day and to increase robustness of the comparison made to the low and high *T*_*CMB*_ mantle-side heat flux models. The calculated q_a_ between 3–4.5 Ga is shown in Fig. 4, with an upper-bound defined by α_c_ = 10.3 10^–5^ K^−1^, C_P_ = 800 J kg^−1^ K^−1^, and high T_CMB_ model, and a lower-bound defined by α_c_ = 5.25 10^–5^ K^−1^, C_P_ = 850 J kg^−1^ K^−1^, and low T_CMB_ model. For comparison, we show recalculated results for the range of Fe-Si compositions from Berrada et al.^46^ when *T*_*CMB*_ and κ_e_ are allowed to vary as described above.

The modified core-side q_a_ Fe–Si band is thicker than the Fe_83_Si_14_S_3_ band since it spans greater range of LE contents. In addition to the q_a_ bound parameters described previously, the upper- and lower-bounds of this band are defined by Fe_98_Si_2_ and Fe_83_Si_17_, respectively. Since κ_e_ ∝ *T* via the WFL and q_a_ ∝ *T*^2^ via Eq. (2), and *T*_*CMB*_ and κ_e_ (*T*_*CMB*_) were allowed to vary, a lunar age-dependence of q_a_ is well defined and an Fe-Si lunar core spans a larger range of core-side q_a_ values (~ 1.0–4.4 mW/m^2^) from 4.5 Ga to present-day than previously determined (~ 1.1–3.3 mW/m^2^)^46^. As shown in Fig. 4, the age of thermal convection shutdown for a Fe_98_Si_2_–Fe_83_Si_17_ lunar core is marginally earlier, from their initial estimate of ~ 3.32–3.80 Ga to ~ 3.38–3.86 Ga using our variable *T*_*CMB*_ and κ_e_ formulation. In contrast, the core-side q_a_ values up to present-day for an Fe_83_Si_14_S_3_ lunar core range from 0.5 to 1.5 mW/m^2^, with thermal convection shutdown estimates of ~ 3.17–3.72 Ga. In both cases, both lunar core compositions appear generally compatible with the estimated duration of a high intensity magnetic field (3.5–4.2 Ga)^32–34^; however, an Fe_83_Si_14_S_3_ lunar core is more constrained to be compatible. In particular, this lunar core composition requires a T_CMB_ that is sufficiently high and a mantle-side heat flux that decreases at a high rate with age. If these conditions are not met, thermal convection ceases well outside the high intensity field estimates, suggesting that the lunar core cannot be comprised of this composition. With an Fe–Si lunar core, greater variability of *T*_*CMB*_ and mantle-side heat flux are permitted that maintain agreement between shutdown ages and high intensity field estimates. However, high Si LE content in Fe-Si alloys follows similar constraints and requirements as an Fe_83_Si_14_S_3_ core composition to remain compatible. A systematic investigation of Fe–Si–S system with low total LE contents is necessary to determine lunar compositions of equal or better compatibility than an Fe-Si composition.

A lunar core containing both S and Si as LEs may be preferable to one solely containing Si since the presence of S decreases both the solidus and liquidus *T* to a greater extent than Si^39,40^. This allows for a completely molten core for a prolonged duration during the high *T* conditions of lunar formation and can permit a partially molten core in the present-day (i.e. solid inner core and liquid outer core). Additionally, as the lunar core cools below the liquidus *T*, solid Fe-Si precipitates out of the molten mixture^39^. Expected to be denser than the residual S-rich liquid, a density-driven convection mechanism may be established as the precipitate sinks towards the center of the core, analogous to Fe snowing inner core formation suggested to occur in the cores of Ganymede, Mercury, and Mars^55^. This may be one mechanism that allowed continued stirring of the lunar outer core to occur well after the high intensity era, alongside other dynamo mechanisms such as mantle precession^36^. Moreover, a density-driven convection mechanism involving a buoyant S-rich liquid phase is consistent with the recent study by Bercovici et al.^56^. They suggested that formation of a molten core in terrestrial bodies accreted from chondritic precursors, such as the moon and its proposed pre-giant impactor Theia (e.g.,^57^), may contain ≥ 5 wt.% S after accounting for loss or removal of immiscible S-rich liquids in differentiated parent bodies of iron meteorites.

## Methods

**Figure 5:**
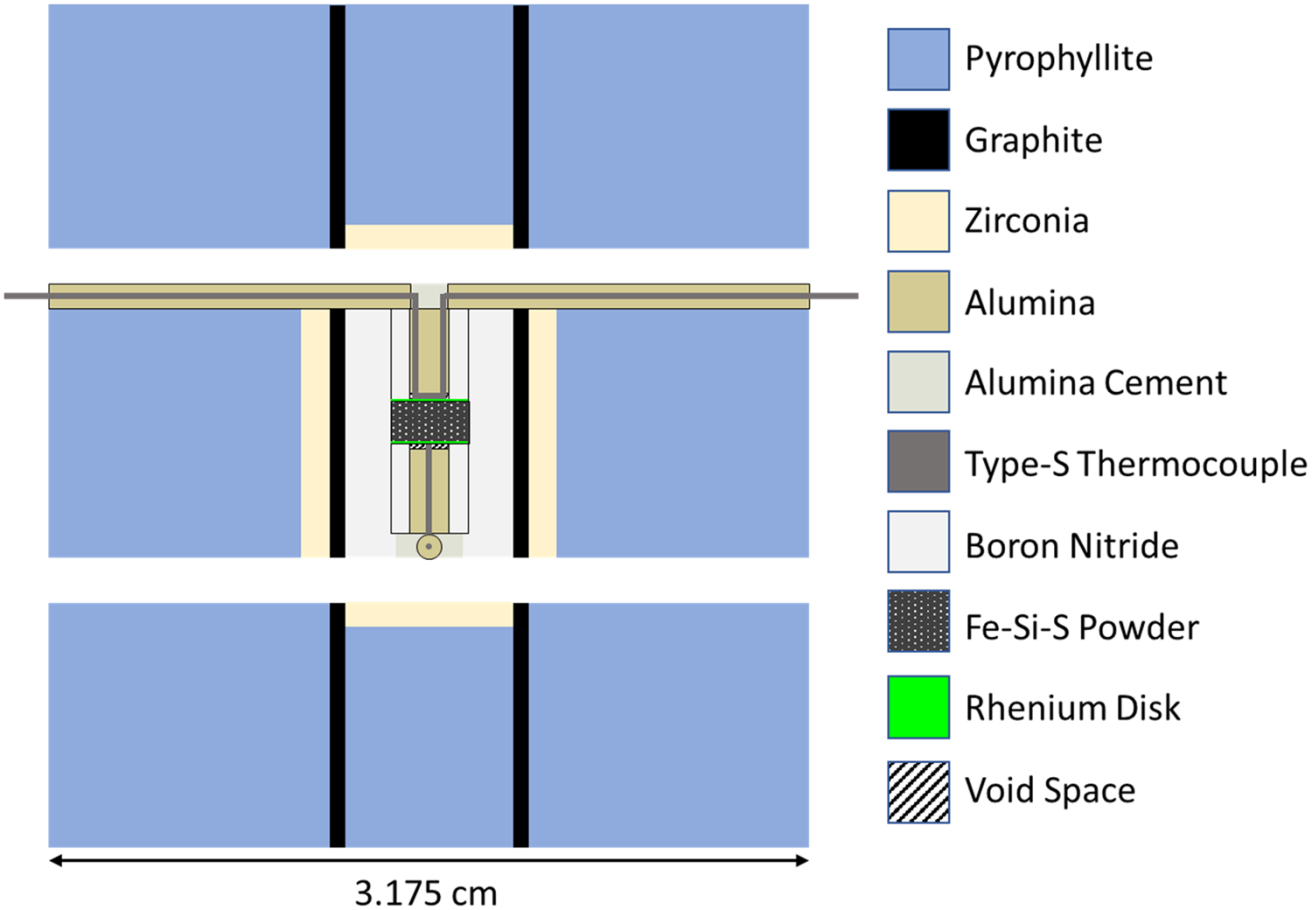
Illustration of the cross-section of the cubic pressure cell used in all experiments.

High purity FeS (99.98%) and Fe_83_Si_17_ (99.5%) powders were purchased from Alfa Aesar and GoodFellow Inc., respectively. The powders were mechanically mixed in proportion (~ 1:8) to obtain a composition of approximately Fe_83_Si_14_S_3_. All experiments were conducted in a 1000-ton cubic anvil press to generate high quasi-hydrostatic *P*. An illustration of the cross-section of the three-sectioned cubic *P* cell design used for all experiments in this study is shown in Fig. 5. The cell design differed from those used previously in Fe–FeS investigations^3,22^ in two ways: (i) utilized Re disks instead of tungsten (W); and (ii) sample diameter was increased to ~ 3.25 mm from ~ 2.79 mm.

A pair of Type-S (platinum (Pt)—platinum–rhodium (Pt–Rh)) thermocouples (TCs) were used to monitor the *T* of the sample. Additionally, the TCs were in mechanical contact with the Re disks that were placed on both circular end faces of the powder sample mixture. A four-wire resistance technique and Keysight 34470A data acquisition meter operating at 20 Hz with a 1 μV resolution were used to measure sample voltage (*V*). The contacts between the TCs and circular faces of the disk-enclosed sample allowed the TCs to serve as electrodes. A constant direct current (*I*) of 0.25 A provided by a Keysight B2961 power source was passed through a pair of neighbouring anvils to the Pt leads while the *V* across the Re disks and sample was measured using the Pt–Rh leads contacting a different pair of lateral anvils. A larger *I* compared to previous studies using a similar design and setup (0.2 A; e.g.,^3,22^) was necessary to increase the signal-to-noise ratio of the measured *V* due to a more electrically conductive (i.e. more Fe-rich) sample material and larger sample diameter. Moreover, the larger disk diameter reduced the *V* contribution by the Re disks and counteracted the larger ρ of Re compared to W at all experimental *P* and *T* conditions. However, the contribution to the sample *V* by the Re disks remained non-negligible and were accounted for^47^.

A high alternating current with a typical amplitude of 350 A was passed through the vertical anvils to the segmented graphite sleeve furnace to generate high *T*. A mechanical switch was used to alternate between *V* measurements of *T* using the TCs and the sample *V* using the four-wire technique. A *P*-correction was made to *V* measured by the TCs while switched to *T* mode. A current polarity switch was used while measuring sample *V* to remove thermoelectric and other parasitic voltage effects. The sample container, boron nitride, is an electrical insulator (~ 10^11^ Ω∙cm) that contributes a negligible *V* and was ignored. A minimum of 10 sample *T* measurements were made immediately prior to and after each sample *V* measurement. At low and relatively high *T*, where the sample was solid, a minimum of 20 sample *V* measurements were made—10 measurements per polarity. At high *T* before anticipated melting, where both measurement and heating rate was increased, a minimum of 10 sample *V* measurements were made—5 measurements per polarity. An increased heating rate was necessary to reduce the duration of Re disk exposure to a molten sample and thus reduce chemical contamination of the sample by the disks.

A Nikon SMZ800 microscope operating at 40 × magnification was used to measure the post-experiment sample diameter (*D*) and length (*L*) after grinding and polishing. Sample geometry is related to ρ through equating Ohm’s Law to Pouillet’s Law, which gives (Eq. 3):

3 $$\uprho = \,\uppi VD^{{2}} /{4}IL$$

where the combined terms of π*D*^2^/4 reflect the circular cross-sectional area of a cylindrically shaped sample. Standard error propagation methods were used to assess the uncertainties of ρ arising from sample *V* fluctuations, contributions and corrections to sample *V* by the Re disks, and microscope and software calibration uncertainty of *D* and *L* measurements. Similar error assessment was used for measurements of *T* along with an additional contribution of a fixed ± 10 K to account for the thermal gradient between the off-center TC junction positions and center of the sample^58^. A JEOL JXA-8530F field-emission electron microprobe operating with a 15 kV acceleration voltage, 60 nA probe current, and 10 μm spot-size beam was used to determine post-experiment sample composition and extent of sample contamination by Re.

## Supplementary Information

Supplementary Information.

## Data Availability

All experimental data are available at http://dx.doi.org/10.17632/3cx762xt62.1.
