# Supplementary material for: Electrical resistivity of the Fe–Si–S ternary system: implications for timing of thermal convection shutdown in the lunar core
Source: Sci Rep. 2022 Nov 8;12:19031. doi: 10.1038/s41598-022-21904-y (PMC9643352; doi:10.1038/s41598-022-21904-y)
Supplement: Supplementary file 1 — Supplementary Information. [file 41598_2022_21904_MOESM1_ESM.pdf]

Scientific Reports

Supplementary Information for

Electrical Resistivity of the Fe-Si-S Ternary System: Implications for Timing of Thermal Convection Shutdown in the Lunar Core

Joshua A.H. Littleton[1,2], Wenjun Yong[1], Richard A. Secco[1]

[1]Department of Earth Sciences, University of Western Ontario, London, ON, Canada N6A5B7

[2]Department of Earth and Planetary Sciences, Washington University in St. Louis, St. Louis, MO, United States, 63130

File Contents

Figure S1: Post-Experiment Sample Cross-Section and Electron Microprobe Results

Figure S2: Phase Diagram and Eutectic Pressure-Dependence of Fe-FeS System

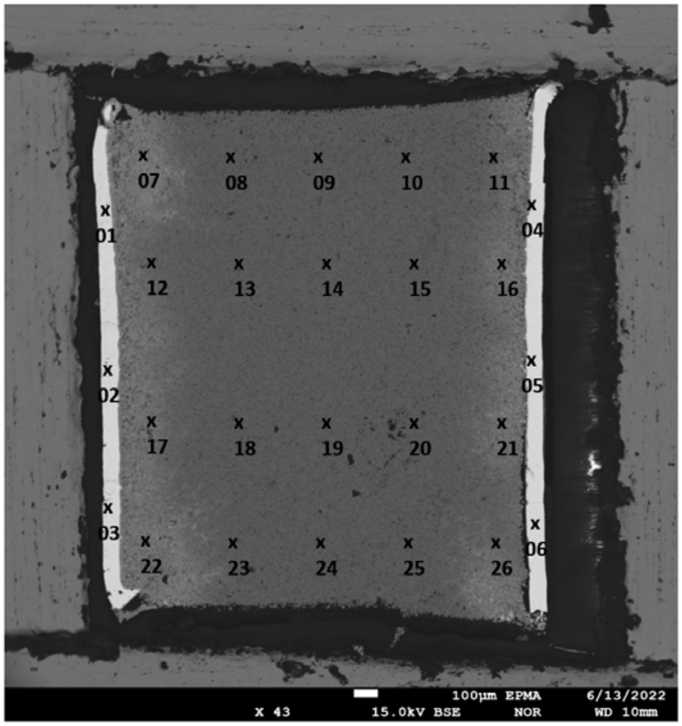

Point #	Fe (wt.%)	Si (wt.%)	S (wt.%)	Re (wt.%)	Pt (wt.%)	Rh (wt.%)	Total (wt.%)
1	0.00	0.14	0.00	97.82	0.07	0.00	98.03
2	0.00	0.14	0.00	100.68	0.00	0.00	100.87
3	0.00	0.14	0.00	100.16	0.00	0.00	100.30
4	0.00	0.13	0.00	101.54	0.03	0.00	101.70
5	0.00	0.15	0.00	101.38	0.06	0.00	101.58
6	0.00	0.14	0.00	102.16	0.07	0.00	102.37
7	61.95	9.65	1.28	27.17	0.00	0.00	100.05
8	82.66	13.25	3.65	0.00	0.00	0.00	99.57
9	84.55	13.17	2.90	0.00	0.00	0.00	100.63
10	85.29	13.04	2.89	0.00	0.00	0.00	101.22
11	75.32	12.79	0.08	11.34	0.00	0.00	99.52
12	76.02	13.14	1.38	9.37	0.00	0.00	99.91
13	82.10	12.46	5.16	0.00	0.00	0.00	99.71
14	83.95	14.14	1.85	0.00	0.00	0.00	99.93
15	82.23	13.01	4.25	0.00	0.00	0.00	99.49
16	67.53	11.13	1.35	20.40	0.00	0.00	100.40
17	65.72	10.30	1.27	23.76	0.00	0.00	101.05
18	83.19	14.25	2.10	0.00	0.00	0.00	99.54
19	86.27	14.21	0.44	0.00	0.00	0.00	100.92
20	84.33	14.62	0.86	0.00	0.00	0.00	99.81
21	66.07	10.45	3.30	19.67	0.00	0.00	99.49
22	67.38	11.67	2.03	17.73	0.00	0.00	98.81
23	83.32	13.51	3.11	0.00	0.00	0.00	99.94
24	78.66	14.47	4.17	0.00	0.00	0.00	97.30
25	83.81	12.39	4.77	0.00	0.00	0.00	100.97
26	76.09	12.30	2.08	10.09	0.00	0.00	100.55

Figure S1: Backscattered electron image of a cross-section of a post-experiment 4 GPa pressure cell centered on the $Fe_{83}Si_{14}S_3$ sample quenched at ~1650 K with annotated microprobe locations. Results of the microprobe have been tabulated.

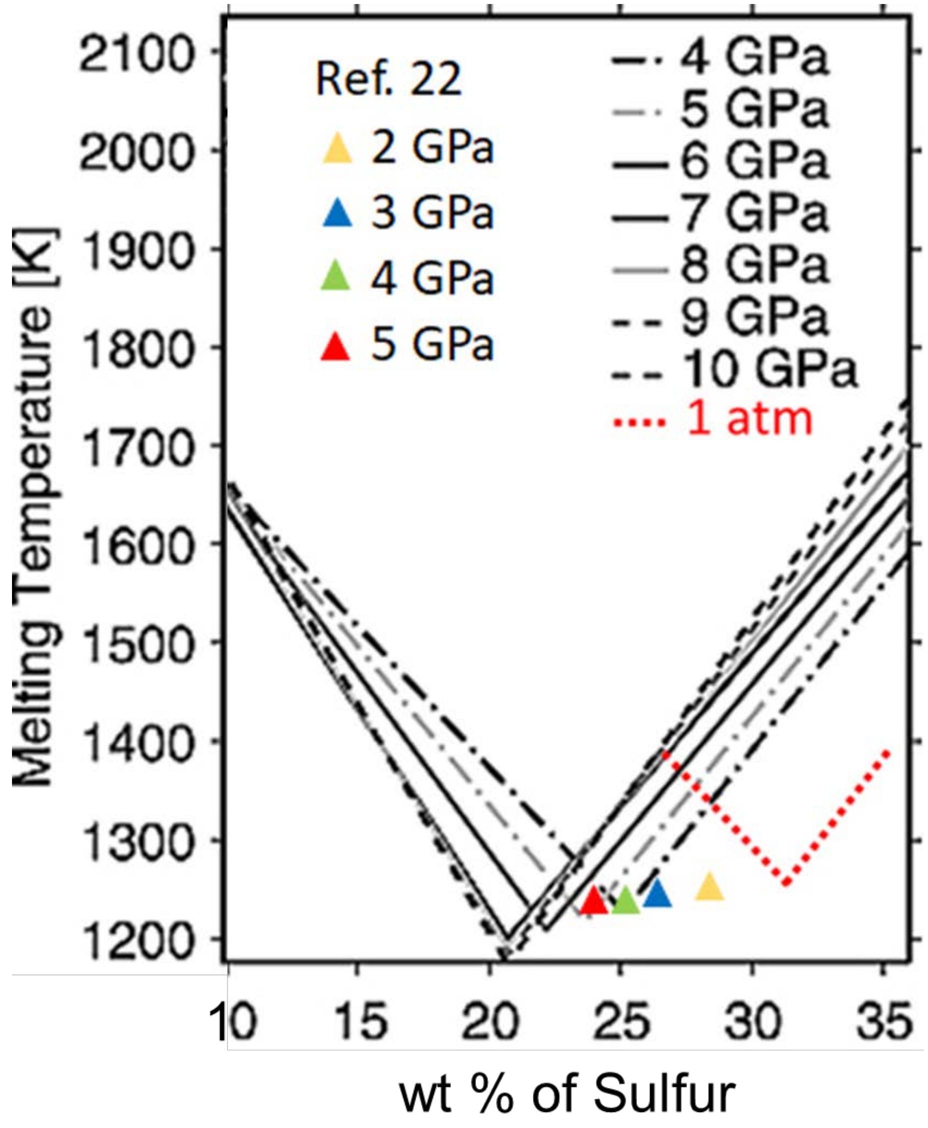

Figure S2: Pressure-dependence of the eutectic and liquidus in the Fe-FeS system modified from a modeling study of Hauck et al. (2006) shown by the lines along with experimentally measured eutectic compositions at pressures of 2-5 GPa[22].

References

Hauck, S.A., Aurnou, J.M. & Dombard, A.J. Sulfur's impact on core evolution and magnetic field generation on Ganymede. *J*. *Geophys*. *Res*.: *Planet*. **11**, 1-14 (2006).
